# Supplementary material for: Uniformity of Food Protein Interpretation Amongst Dietitians for Patients with Phenylketonuria (PKU): 2020 UK National Consensus Statements
Source: Nutrients. 2020 Jul 24;12(8):2205. doi: 10.3390/nu12082205 (PMC7468820; doi:10.3390/nu12082205)
Supplement: Supplementary file 1 [file nutrients-12-02205-s001.pdf]

APPENDIX A:
LOW PROTEIN LABELLING CONSENSUS STATEMENTS FOR PEOPLE WITH PKU ON ≤10G/DAY OF NATURAL PROTEIN

Foods best avoided

FOOD GROUPS	STATEMENTS	% agreement
Meat (lamb, beef, pork, ham, bacon, chicken. turkey, duck, game, beef jerky, sausages, beef or chicken burgers, meat paste, meat pies). Offal (liver, kidney, tongue). Canned meats (e.g. corned beef)	High in protein and are best avoided.	83
Fish (all varieties including shellfish, frozen or tinned fish)	High in protein and are best avoided.	83
Eggs – boiled, poached, fried, scrambled, omelette	High in protein and are best avoided.<br>However, any foods with eggs as an ingredient (e.g. cakes or sweets), must be calculated/weighed as an exchange food if they contain protein >0.5g/100g.	100
Cheeses (all dairy based cheese and cheese spreads)	High in protein and are best avoided.<br>However, any foods with cheese as an ingredient (e.g. in baby foods or sauce), must be calculated/weighed as an exchange food if they contain protein >0.5g/100g.	100
Nuts	High in protein and are best avoided.<br>**Exception:** whole chestnuts	82
Aspartame (E951) or neotame containing food and drinks	All foods, drinks and tabletop sweeteners containing aspartame or neotame are best avoided.	100
Seeds	High in protein and so are best avoided.<br>However, any food products with seeds as an ingredient (e.g. cereal bar) must be calculated/weighed as an exchange food if they contain protein >0.5g/100g.	100
Soya based foods	High in protein and are best avoided.	100
Tofu	High in protein and best avoided.	100
Quorn based foods	High in protein and are best avoided.	100
Goji berries	High in protein and are best avoided.	100
Yeast extract spreads	High in protein and are best avoided.	90
Pork crackles/pork scratching	High in protein and are best avoided.	82

Exchange-free foods

Definition: Any food given without protein measurement/restriction is referred to as an ***exchange-free food.***

FOOD GROUPS	STATEMENTS	% agreement
Fresh, canned, frozen or dried fruit & vegetables ≤75mg Phe/100g	Fruits and vegetables (except potatoes) containing a Phe content **≤75 mg/100g** weight of fruit & vegetable are exchange-free.	100
Sugar – granulated, white, brown, demerara, muscovado, castor, icing sugar, glucose, fructose, lactose, maltose, maltodextrin	Exchange-free **excluding tabletop sugars containing aspartame.**	100
Syrup – golden, maple, agave, flavoured syrups	Exchange-free if they contain exchange-free ingredients only.	100
Black Treacle	Exchange-free as it contains exchange-free ingredients only.<br>The protein content appears higher than other sugars due to its processing. Treacle is made from the 'remaining' syrup after sugar is refined.	100
Fruit syrups/fruit sauces	Exchange-free if they contain exchange-free ingredients only.	100
Sweet spreads e.g. Jam/marmalade/honey	Exchange-free if they contain exchange-free ingredients only.	100
Artificial sweeteners e.g. acesulfame, cyclamate, erythritol, fructose, isomalt, lactitol, maltitol, maltodextrin, mannitol, saccharin, sorbitol, stevia, sucralose, thaumatin, xylitol	All artificial sweeteners/sugar substitute **except aspartame or neotame**, are exchange-free.<br>Aspartame and neotame are best avoided.	96
Fats (butter, margarine, low fat spreads, oils, vegetable fats, oil sprays)	Exchange-free, irrespective of the protein content on the food ingredient label, due to the small quantities consumed.	96
Vinegar-based sauces: Worcester sauce, mint sauce/jelly, vinegar	Exchange-free if they contain exchange-free ingredients only or are used in small quantities.	80
Natural fruit juice (no added sugar)	Exchange- free if they contain exchange-free/aspartame-free ingredients only.	100
Fruit smoothie	Exchange-free if made with exchange-free, aspartame-free fruit/vegetable juice.	100
Soft drinks e.g. squash, fizzy drinks, coke	Exchange-free, providing aspartame-free.	100
Sugar-free drinks e.g. squash, fizzy drinks, cola	Exchange-free, providing aspartame-free.	100
Tea - black, green, mint, camomile, fruit, herbal, and iced.	Exchange-free, providing aspartame-free.	100
Chewing gum/Bubble gum	Chewing gum/bubble gum that is aspartame-free is exchange-free. Chewing gum that contains aspartame is best avoided.	95
Coffee - black ground, instant, expresso	Exchange-free if it contains exchange-free ingredients only.<br>White coffee (including dried reconstituted from sachets), frappuccino's, cappuccino, lattes, coffee pods/capsules with added milk, iced coffee, macchiato must be calculated/measured as exchange drinks.	100
Herbs, spices, seasonings (fresh or dried), salt & pepper	All herbs, spices and seasonings fresh or dried (irrespective of the protein content on the food ingredient label) are exchange-free, due to the small quantities consumed.	100

Custard powder containing exchange-free ingredients only	Custard powders containing exchange-free ingredients only (e.g. cornflour, colour, flavourings) are exchange-free. **Prepare with protein-free milk.**	100
Milkshake powders/syrups containing exchange-free ingredients and aspartame-free.	Milkshake syrups or powders containing exchange-free ingredients only (e.g. cornflour, colour, flavourings) are exchange-free. **Prepare with protein-free milk.**	100
Food colouring (gel, liquid or paste) and flavouring/essences (e.g. vanilla, peppermint, almond)	Exchange-free if they contain exchange-free ingredients only.	100
Flours - arrowroot, cornflour/maize starch/corn starch, cassava/tapioca starch, sago	Exchange-free.<br>Note: Cornmeal is high in protein and is an exchange food. Ready-to-serve milk puddings made with tapioca and sago are not exchange-free.	100
Baking ingredients – bicarbonate of soda, baking powder, cream of tartar	Exchange-free.	100
Fibres/gums – psyllium fibre, psyllium husks, xanthan gum	Exchange-free.	100
Konnyaku	Exchange-free.	100
Cassava crisps	Exchange-free if ingredients are all exchange-free.	100

Foods that require measuring/weighing as an exchange food if they contain protein >0.1g/100g or 0.1g/100ml. If they have a protein content no more than 0.1g/100g or 0.1g/100ml, they are exchange-free.

FOOD GROUPS	STATEMENTS	% agreement
Milk (animal based)– full fat, semi-skimmed, skimmed, condensed, soya	Any milk product (plant or animal) containing protein >0.1g /100ml must be measured/calculated as an exchange food.	80
Plant milks e.g. coconut, oat, rice, almond, hemp<br>Includes coffee with these added e.g. dried reconstituted from sachets, frappuccino's, cappuccino, lattes, coffee pods/capsules with added milk, iced coffee, macchiato	Any milk product (plant or animal) containing protein >0.1g /100ml must be measured/calculated as an exchange food.<br>Coffee either containing or made with animal/plant milk must be calculated/measured as an exchange drink. Exchange-free if it contains exchange-free ingredients only.	80

Foods that require measuring/weighing as an exchange food if they contain protein >0.5g/100g. If they contain exchange-free ingredients only or have a protein content no more than 0.5g/100g, they are exchange-free.

FOOD GROUPS	STATEMENTS	% agreement
**General food groups**		
Gluten-free foods	Gluten-free foods with a protein content >0.5g/100g must be calculated/weighed as exchange foods.	96
Gelatine containing foods	All foods containing gelatine and with a protein content >0.5g/100g must be calculated/weighed as exchange foods.	93
**Dairy products and alternatives**		
Vegan, free-from or soya/plant cheese or regular cheese as an ingredient	Any foods with added regular cheese as an ingredient (e.g. in baby foods or sauce), and with a protein content >0.5g/100g, must be calculated/weighed as an exchange food.<br><br>Any vegan or 'free-from' cheese is exchange-free if the protein content is ≤0.5 g/100g or if they contain exchange-free ingredients only. Any with exchange ingredients and a protein content >0.5g/100g must be calculated/weighed as an exchange food.	94
Soft cheese, cheese spread, cream cheese	Soft, cream cheeses with a protein content >0.5g/100g must be calculated/weighed as an exchange food.<br><br>Any vegan or free-from soft cheese is exchange-free if the protein content is ≤0.5 g/100g or if they contain exchange-free ingredients only. If they contain protein >0.5g/100g and contain exchange ingredients, they must be calculated/weighed as exchange foods.	87
Ice-lolly milk based	Exchange-free if the protein content is ≤0.5g/100g of food. Any with exchange ingredients and a protein content >0.5g/100g must be calculated/weighed as an exchange food.	96
Ice-lolly fruit/juice based	Exchange-free if the protein content is ≤ 0.5g/100 of food. Any with exchange ingredients and a protein content >0.5g/100g must be calculated/weighed as an exchange food.	96
Cream e.g. double, single, clotted, cream alternatives, sour, spray cream	Any cream with a protein content >0.5g/100g must be calculated/weighed as an exchange food.	100
Ice-cream (dairy and dairy-free)	Any ice-cream (dairy or dairy-free) with a protein content >0.5g/100g must be calculated/ weighed as an exchange food.<br><br>**Weight** rather than the volume of ice-cream must be used to calculate the protein exchange amount.	100
Dairy or soya yogurt e.g. flavoured or plain, drinking, soya	Any yoghurts with a protein content >0.5g/100g must be calculated/weighed as an exchange food.<br><br>However, if they contain exchange ingredients and protein ≤0.5g/100g, it is best to limit to 1 portion/pot per day.	100
Dairy desserts e.g. fromage frais, dairy desserts, custard, mousse, instant dessert, rice pudding	Any desserts with a protein content >0.5g/100g must be calculated/weighed as an exchange food.<br><br>However, if they contain exchange ingredients and protein ≤0.5g/100g, it is best to limit to 1 portion/pot per day.	97

Coconut-based desserts	Any coconut desserts with a protein content >0.5g/100g must be calculated/weighed as an exchange food. If they contain ≤0.5g/100g, they are considered exchange-free.<br><br>However, if they contain exchange ingredients and protein ≤0.5g/100g, it is best to limit to 1 portion/pot per day.	97
Coconut products	Dried coconut products with a protein content >0.5g/100g protein must be calculated/weighed as an exchange food.	93
**Breads & cereals**		
Bread e.g. bread roll, English muffin, bagels, crumpet, pitta, naan, ciabatta, wrap, breadsticks, croissants, brioche (including gluten-free)	All breads and bread products, including gluten-free varieties, with a protein content >0.5g/100g must be calculated/weighed as an exchange food.	100
Breakfast cereals – **wheat based** e.g. muesli branflakes, multigrain, shredded wheat, Weetabix and **corn, rice or oat based** e.g. Cornflakes, Rice krispies, Cheerios, Sugar puffs, Frosties, oats/porridge (cooked), including gluten-free	All breakfast cereals and breakfast oats (including gluten-free varieties) with a protein content >0.5g/100g must be calculated/weighed as an exchange food.	100
Cereal grains e.g. wheat, bulgar wheat, quinoa, barley, oats, corn, rice, semolina, millet sorghum, buckwheat, quinoa, chia, triticale, amaranth	All cereals with a protein content >0.5g/100g must be calculated/weighed as exchange foods.	100
Yorkshire pudding, stuffing balls, pancakes, waffles (including gluten-free)	All Yorkshire puddings, pancakes, waffles, stuffing balls, (including gluten-free varieties) with a protein content >0.5g/100g must be calculated/weighed as an exchange food.	100
**Spreads & dips**		
Lemon curd	All lemon curd with a protein content >0.5g/100g must be calculated/weighed as an exchange food.	100
Chocolate spread	All chocolate spread with a protein content >0.5g/100g must be calculated/weighed as an exchange food.	100
Nut spread/chocolate nut spread e.g. peanut butter, Nutella	All nut spreads with a protein content >0.5g/100g must be calculated/weighed as an exchange food.	87
Hummus	Hummus with a protein content >0.5g/100g must be calculated/weighed as an exchange food.	100
Dips – sweet and savoury	Any sweet or savoury dips (e.g. sour cream, Tzatziki, nacho cheese dip) require measuring/weighing as exchange foods if they contain protein >0.5g/100g and contain exchange ingredients. If they contain exchange-free ingredients only or have a protein content no more than 0.5g/100g, they are exchange-free.	100
**Sauces, dips, gravy & soup**		
Gravy	Exchange-free if the protein content is **≤ 0.5 g/100 ml when prepared.** Any with exchange ingredients and a protein content >0.5g/100g must be calculated/measured as an exchange food.	100
Cheese or milk-based sauces e.g. cheese, white, parsley, carbonara	All cheese sauces must be calculated/measured as part of exchange system unless dry powder ingredients are exchange-free and are made up with water or protein-free milk.	100
Herby/spicy rubs or coatings	Exchange-free if the protein content is ≤0.5g per serving. Any that contain exchange ingredients with a protein content >0.5g/100g per serving must be calculated/weighed as an exchange food.	96

Pesto, mustard, tapenade, plant-based vegetable spreads	Any pesto, mustard, tapenade or plant-based spread, including homemade, with a protein content >0.5g/100g must be calculated/weighed as an exchange food (unless it contains exchange-free ingredients only).	100
Soup	If soups contain exchange ingredients and their protein content is >0.5 g/100 g, then they must be calculated/measured as an exchange food (unless they contain exchange-free ingredients only).	79
**Pasta & rice**		
Regular & gluten-free pasta, rice, noodles, couscous, gnocchi, tinned pasta, pasta shapes	All regular or gluten-free pasta, rice or pasta products with a protein content >0.5g/100g must be calculated/weighed as an exchange food.	100
Pot Noodles	All pot noodles with a protein content >0.5g/100g when reconstituted, must be calculated/weighed as an exchange food.	100
**Meat & Alternatives**		
Vegan meat e.g. vegetarian burger/sausages, *Hooba* mushroom sausages	Any vegan meat replacements must be calculated/weighed as an exchange food if it contains protein >0.5g/100g and contains protein exchange ingredients.	83
Vegan fish	Any vegan fish replacements must be calculated/weighed as an exchange food if it contains protein >0.5g/100g and contains protein exchange ingredients.	83
Vegan Eggs, including aquafaba	Any vegan egg replacements must be calculated/weighed as an exchange if it contains protein >0.5g/100g and contains protein exchange ingredients.	100
Legumes/pulses e.g. Lentils, chickpeas, dried peas, split peas. Beans – baked, red & black, kidney, haricot, pinto, cannellini, turtle, butter, adzuki	All pulses and beans with a protein content >0.5g/100g must be calculated/weighed as an exchange food. **Exception**: French green beans are exchange-free.	95
**Drinks**		
Hot chocolate, malted milk powders, milkshake powders or liquids	Flavoured milk powders, milkshake powders/liquids and hot chocolate powders/liquids with a protein content >0.5g/100g must be calculated/measured as an exchange food. These are best prepared with protein-free milk.	94
**Sweet snacks**		
Biscuit/cookie	Biscuits with a protein content >0.5g/100g must be calculated/weighed as an exchange food.	100
Cake, muffin, doughnut, tart, scone, pastries, brownies, flapjack, fruit loaves, fruit pies, scones, cupcakes, cake bars, teacakes (including gluten-free varieties).	Cakes and pastries, including gluten-free varieties, with a protein content >0.5g/100g must be calculated/weighed as an exchange food.	100
Breakfast or cereal bars	Breakfast and cereal bars with a protein content >0.5g/100g must be calculated/weighed as an exchange food.	100
Fruit bars	Any fruit bars with a protein content >0.5g/100g must be calculated/weighed as an exchange food (unless they contain exchange-free ingredients only).	97
Puddings – cheesecake, crumble, pie, tart, sponge, trifle, meringue, sponge	Any desserts with a protein content >0.5g/100g must be calculated/weighed as an exchange food.	100
Frozen puddings e.g. pies, pastries, crumbles (including gluten-free varieties).	All frozen pastry, pies, crumbles (including gluten-free varieties) with a protein content >0.5g/100g must be calculated/weighed as an exchange food.	100

Jelly (aspartame-free)	Exchange-free if the protein content is ≤0.5g/100g. Any with exchange ingredients and a protein content >0.5g/100g must be calculated/weighed as an exchange food.	97
Chocolate/chocolate bars, fudge, toffee	Exchange-free if the protein content is ≤0.5g/100g**.** All with exchange ingredients and a protein content >0.5g/100g must be calculated/weighed as an exchange food.	100
Gummy sweets, vegetarian gummy sweets (aspartame-free)	Exchange-free if the protein content is ≤0.5g/100g. Any with exchange ingredients and a protein content >0.5g/100g must be calculated/weighed as an exchange food.	91
Marshmallows	Exchange-free if the protein content is ≤0.5g/100g. Any with exchange ingredients and a protein content >0.5g/100g must be calculated/weighed as exchange foods.<br>**Note:** only gelatine-free mallows are likely to contain ≤0.5g/100g protein.	95
All other sweets e.g. boiled, chewy, mints, candy (aspartame-free)	Exchange-free if the protein content is ≤0.5g/100g. Any with exchange ingredients and a protein content >0.5g/100g must be calculated/weighed as an exchange food.	91
Liquorice	Liquorice with a protein content >0.5g/100g must be calculated/weighed as an exchange food.	100
**Savoury snacks**		
Potato crisps	All potato crisps with a protein content >0.5g/100g must be calculated/weighed as an exchange food.	100
Vegetables crisps	All vegetable crisps with a protein content >0.5g/100g must be calculated/weighed as an exchange food.<br>**Exceptions**: Cassava crisps are exchange-free if ingredients are all exchange-free.<br>Vegetable crisps containing exchange-free vegetables are calculated/weighed as exchange foods due to the concentration of protein associated with cooking methods; their protein content per 100g must be used to determine the exchange amount.	100
Corn and rice-based snacks	All corn and rice-based snacks with a protein content >0.5g/100g must be calculated/weighed as an exchange food.	100
Popcorn	All popcorn with a protein content >0.5g/100g must be calculated/weighed as an exchange food.	100
Pretzels	All pretzels with a protein content >0.5g/100g must be calculated/weighed as an exchange food.	100
Crackers, crispbread, breadsticks	Crackers, crispbreads and breadsticks with a protein content >0.5g/100g must be calculated/weighed as an exchange food.	100
Rice, corn or oat cakes	Rice, corn or oat cakes with a protein content >0.5g/100g must be calculated/weighed as an exchange food.	100
**Flours and Starch**		
Flour and starch e.g. white, self-raising, wholemeal, chapatti, bread mixes, gram flour, gluten-free, coconut flour, rye flour, spelt flour, almond flour, nut flours, rice flour, peasemeal, chestnut flour, sorghum flour, atta flour, maida flour, spelt flour, cornmeal	All flours with a protein content >0.5g/100g must be calculated/weighed as an exchange food.	100
**Sugars**		

Icing – e.g. Buttercream, fondant icing	All icings with a protein content ≤0.5g/100g or if they contain exchange-free ingredients only, are considered exchange-free. Any with exchange ingredients and a protein content >0.5g/100g must be calculated/weighed as an exchange food.	100
Marzipan	Marzipan with a protein content >0.5g/100g must be calculated/weighed as an exchange food	100

Foods that require measuring/weighing as an exchange food if they contain protein >1g/100g. If they contain exchange-free ingredients only, or have a protein content no more than 1g/100g, they are exchange-free.

FOOD GROUPS	STATEMENTS	% agreement
**Sauces & dressings**		
Table top sauces, brown sauce, tomato ketchup, BBQ, chilli, fat-free dressings, chutney, pickles, mint sauce, salad dressings, vinaigrette, Horseradish, and tartare.	Require measuring/weighing as exchange foods if they contain protein >1g/100g and contain exchange ingredients. If they contain exchange-free ingredients only or have a protein content no more than 1g/100g, they are exchange-free.	80
Mayonnaise, salad cream, oil-based dressings	Require measuring/weighing as exchange foods if they contain protein >1g/100g and contain exchange ingredients. If they contain exchange-free ingredients only or have a protein content no more than 1g/100g, they are exchange-free.	91
Cook-in/pour over or liquid sauces e.g. Italian, curry, sweet & sour, tomato, vegetable	Require measuring/weighing as exchange foods if they contain protein >1g/100g and contain exchange ingredients. If they contain exchange-free ingredients only or have a protein content no more than 1g/100g, they are exchange-free.	87
**Sugars**		
Cake decorations	All cake decorations are exchange-free if they contain exchange-free ingredients only or if the protein content is ≤1g/100g. If they contain >1g/100g and contain exchange ingredients, they must be calculated/weighed as exchange foods.	97

Foods that require measuring/weighing as an exchange food if they contain protein >1.5g/100g. If they contain exchange-free ingredients only or have a protein content no more than 1.5g/100g, they are exchange-free.

FOOD GROUPS	STATEMENTS	% agreement
Soya sauce	Exchange-free if it contains protein ≤1.5g/100g. Any with a protein content >1.5g/100g must be calculated/measured as an exchange food	79

Special low protein food containing Phe >25 mg/100g and containing exchange ingredients such as milk or seeds

FOOD GROUPS	STATEMENTS	% agreement
Low protein special foods (including bread, bread rolls, baguettes, pizza bases, breakfast cereals, chocolate spread, pasta, noodles, rice, couscous, sausage/burger mix, snack pots, biscuits/cookies, cakes, cereal bars, puddings, chocolate, crackers, sauces, flour, cake mix, dessert mix)	Exchange-free if they contain exchange-free ingredients only.<br>If low protein special prescribed foods contain exchange ingredients but contain Phe **≤25mg Phe/100g** of prepared product, they are exchange-free. If they contain exchange ingredients and contain Phe **≥26/100g** of prepared product, they are an exchange food.	83

Low Protein Milk Replacements

FOOD GROUPS	STATEMENTS	% agreement
Low protein milks replacements (available on prescription)	Any low protein special milk that provides a Phe intake of **>5mg/100ml** must be calculated/measured as an exchange food.	80

Fruit and Vegetables as exchange foods

FOOD GROUPS	STATEMENTS	% agreement
Fresh fruit & vegetables 76-100mg Phe/100g	Fruit and vegetables containing a Phe content **76–99 mg/100g** weight of fruit and vegetable, are calculated/weighed as exchange foods<br><br>A standard exchange amount of **60g** for any fruit or vegetables containing Phe between 76-99 mg/100 g is used.	80
Fresh fruit & vegetables ≥100mg Phe/100g	Fruits and vegetables with a Phe content of **≥100 mg/100g** weight of fruit and vegetable, are calculated/weighed as exchange foods. Their phenylalanine content is used to calculate exchange amounts.	100
Manufactured fruit & vegetables e.g. frozen, canned, passata, tomato puree	Any manufactured fruit or vegetables (e.g. frozen or canned products) that are exchange-free in their fresh form, are exchange-free foods if they contain exchange-free ingredients only, e.g. herbs, oil.<br><br>However, if they contain added ingredients that are protein containing (e.g. milk or wheat), they are calculated/weighed as exchange foods.	96
Dried fruit e.g. apricot, dates, cranberries, banana chips, mango, raisins, sultanas, fruit bars	Any dried fruits that are exchange-free in their fresh form, are exchange-free foods.<br><br>However, if they contain added ingredients that are protein containing (e.g. milk or wheat), they are calculated/weighed as exchange foods.	100
**Potato and potato products**		
Potato - boiled, mashed (no milk), jacket, baked	**80g = 1 exchange of protein, based on phenylalanine analysis.**<br><br>Fresh/frozen potatoes are calculated/weighed as exchange foods. If potato products contain additional exchange ingredients (e.g. wheat flour, or milk), the protein analysis on the packet is used to determine its exchange amount.	96
Canned potato	**100g = 1 exchange of protein, based on phenylalanine analysis.**<br><br>If potato products contain additional exchange ingredients (e.g. wheat flour, or milk), the protein analysis on the packet is used to determine its exchange amount.	96
Roast potato (fresh/frozen)	**55g = 1 exchange of protein, based on phenylalanine analysis.**<br><br>If potato products contain additional exchange ingredients (e.g. wheat flour, or milk), the protein analysis on the packet is used to determine its exchange amount.	96
Chips/fries (fresh/frozen)	**45g = 1 exchange of protein, based on phenylalanine analysis.**<br><br>If potato products contain additional exchange ingredients (e.g. wheat flour, or milk), the protein analysis on the packet is used to determine its exchange amount.	96
Instant mashed potato	**10g (dry weight) = 1 exchange of protein based on phenylalanine analysis.**<br><br>If potato products contain additional exchange ingredients (e.g. wheat flour, or milk), the protein analysis on the packet is used to determine its exchange amount.	96
Processed potatoes – waffles, croquettes, wedges, Alphabites, hash browns, Smiley faces, potato salad	Fresh/frozen potato products are calculated/weighed as exchange foods. If Phe analysis is unavailable, the protein content is used to calculate exchange amounts. If potato products contain additional exchange ingredients (e.g. wheat flour, or milk), the protein analysis on the packet is used to determine its exchange amount.	96

General statements*

	STATEMENTS
Rounding values up or down if protein is <0.5g or >0.5g/100g	**Definition: 1g protein exchange = the amount of food that is calculated/measured to provide 1g protein from the protein food analysis.**<br><br>Food 'exchange' amounts for food portions must be rounded up or down based on the 'rule of maths' (Table 1* below for guidance).
General guidance	Patients/caregivers are advised to read ingredient lists as well as the protein content per 100g on food labels.<br><br>As guidance:<br>- If ingredients are protein containing (but the protein analysis appears very low), it is important to establish more accurate protein analysis before consuming.<br>- If ingredients are 'clearly' exchange-free, then it is appropriate to give as an exchange-free food even if the protein analysis is unavailable on the label.<br>- If there is no nutritional analysis on the product but it contains ingredients that are exchange foods, then further food protein analysis must be obtained before consuming.<br>- If a label states protein content is 0 g but one or more ingredients is a protein source (e.g. gelatine), then an accurate food protein analysis must be obtained before consuming.

*previously reported in: Evans S, Ford S, Adam S, Adams S, Ash J, Ashmore C, et al. Development of national consensus statements on food labelling interpretation and protein allocation in a low phenylalanine diet for PKU. Orphanet J Rare Dis. 2019;14:2.

Table 1: Calculating food protein exchanges from protein analysis on the food label

**Protein content per item when calculated from label**	**Calculated exchange**
0g protein per food portion	Exchange free
0.1g protein per food portion	Exchange free (if total volume consumed is ≤ 0.5g protein)
0.2g protein per food portion	Exchange free (if total volume consumed is ≤ 0.5g protein)
0.3g protein per food portion	Exchange free (if total volume consumed ≤ 0.5g protein).<br><br>Suggest 1 portion is exchange-free, 2 portions is ½ exchange*
0.4 to 0.7g protein per food portion	½ exchange protein
0.8 to 1.2g protein per food portion	1 exchange protein
1.3 to 1.7g per food portion	1.5 exchange protein
1.8 to 2.2g per food portion	2 exchange protein
2.3 to 2.7g per food portion	2.5 exchange protein
2.8 to 3.2 per food portion	3 exchange protein

*This may apply to ice cream lolles, gluten-free cakes, very small packets crisps, sweets
